# Rescue of *Citrus sudden death‐associated virus* in *Nicotiana benthamiana* plants from cloned cDNA: insights into mechanisms of expression of the three capsid proteins

**DOI:** 10.1111/mpp.12780

**Published:** 2019-01-29

**Authors:** Emilyn E. Matsumura, Helvécio D. Coletta‐Filho, Marcos A. Machado, Shahideh Nouri, Bryce W. Falk

**Affiliations:** ^1^ Department of Plant Pathology University of California Davis CA 95616 USA; ^2^ Centro de Citricultura Sylvio Moreira Instituto Agronômico de Campinas Cordeirópolis SP 13490‐970 Brazil; ^3^ Department of Plant Pathology Kansas State University Manhattan KS 66506 USA

**Keywords:** capsid protein expression, citrus disease, *Citrus sudden death‐associated virus*, infectious clone, *Marafivirus*, *Tymoviridae*

## Abstract

*Citrus sudden death‐associated virus* (CSDaV) is a member of the genus *Marafivirus *in the family *Tymoviridae*, and has been associated with citrus sudden death (CSD) disease in Brazil. Difficulties in the purification of CSDaV from infected citrus plants have prevented progress in the investigation of the role of this virus in CSD and an understanding of its molecular biology. In this work, we have constructed a full‐length cDNA clone of CSDaV driven by the 35S promoter (35SRbz‐CSDaV). *Agrobacterium tumefaciens*‐mediated inoculation of 35SRbz‐CSDaV in *Nicotiana benthamiana* plants enabled a fast recovery of large amounts of virions from the agroinfiltrated leaves, which allowed a better molecular characterization of CSDaV. *In vivo* analyses of mutant versions of 35SRbz‐CSDaV revealed the expression strategies used by CSDaV for production of the capsid proteins (CPs). We showed that CSDaV virions contain three forms of CP, each of which is generated from the same coding sequence, but by different mechanisms. The major CPp21 is a product of direct translation by leaky scanning from the second start codon in the subgenomic RNA (sgRNA), whereas the minor CPs, p25 and p23, are produced by direct translation from the first start codon in the sgRNA and by *trans*‐proteolytic cleavage processing derived from the p25 precursor, respectively. Together, these findings contribute to advance our understanding of CSDaV genome expression strategies. In addition, the construction and characterization of the CSDaV infectious clone represent important steps towards the investigation of the role of this virus in CSD and of its use as a tool for citrus biotechnology.

## Introduction

*Citrus sudden death‐associated virus* (CSDaV) is a monopartite, positive‐sense, single‐stranded RNA virus that belongs to the genus *Marafivirus *in the family *Tymoviridae*, and has been associated with citrus sudden death (CSD) disease in Brazil (Maccheroni *et al.*, 2005; Matsumura *et al.*, 2016, 2017). CSD‐affected plants show yellow staining in the rootstock bark and symptoms of general decline, such as pale green coloration of the leaves, overall defoliation and death of the roots (Bassanezi *et al*., 2007; Müller *et al.*, 2002; Román *et al.*, 2004). Between 1999 and 2004, CSD caused the death of approximately four million plants of sweet orange (*Citrus sinensis* L. Osb), mostly grafted on Rangpur lime rootstock (*Citrus limonia *L. Osb), which was, at that time, the main non‐irrigated rootstock used in the most important citrus region in Brazil (Bassanezi *et al*., 2007; Müller *et al.*, 2002; Román *et al.*, 2004). Consequently, CSD led to the replacement of Rangpur lime with CSD‐tolerant rootstocks in this region, which has contributed in preventing the spread of the disease, but, on the down side, has required irrigation systems in order to maintain the productivity achieved with the previously used rootstock.

Almost two decades after CSD’s first detection, its aetiology has still not been definitively determined and the association of CSDaV with CSD remains unclear. Difficulties in purifying CSDaV virions from infected citrus plants and in generating CSD symptoms using conventional methods of transmission, such as rub inoculation, grafting and aphids (Santos, 2011), have prevented progress in the investigation of the role of this virus in CSD. Thus, the construction of a CSDaV infectious clone is essential to advance our knowledge of the molecular biology of this virus. Cloned viral genomes can be easily modified for the functional analysis of viral genes and for a better understanding of RNA virus–vector–plant interactions (Gao *et al.*, 2012; Tuo *et al.*, 2015; Wieczorek *et al.*, 2015).

From what is known so far, CSDaV virions are isometric particles of about 30 nm in diameter and the RNA genome is approximately 6.8 kb in length encompassing two open reading frames (ORFs) (Maccheroni *et al.*, 2005). The larger ORF encodes a 240‐kDa polyprotein (p240), which contains conserved signatures for the methyltransferase (MT), cysteine protease (PRO), helicase (HEL), RNA‐dependent RNA polymerase (RdRP) and capsid protein (CP) domains (Maccheroni *et al.*, 2005). Previous work, largely based on nucleotide sequence comparisons, predicted the production of two subunits of the CP: a major CPp21 (~21 kDa), probably encoded by a subgenomic RNA (sgRNA), and a minor CPp22.5 (~22.5 kDa), which was thought likely to be generated by a proteolytic cleavage of the polyprotein encoded by the genomic RNA (gRNA) (Maccheroni *et al.*, 2005). The small ORF at the 3′ end region seems to encode for a protein of 16 kDa (p16), which, so far, has an unknown function (Maccheroni *et al.*, 2005).

In this work, we have constructed a full‐length cDNA infectious clone of CSDaV (35SRbz‐CSDaV) which can replicate and assemble in *Nicotiana benthamiana *plants. *Agrobacterium tumefaciens*‐mediated inoculation of 35SRbz‐CSDaV induces severe hypersensitive response (HR)‐like necrotic symptoms on *N. benthamiana*‐agroinfiltrated leaves. However, we were able to recover large amounts of virions from the agroinfiltrated leaves just before the HR symptoms appeared (only 2 days after agroinoculation). Such a fast recovery allowed us to quickly analyse, *in vivo*, wild‐type (WT) and mutant versions of 35SRbz‐CSDaV in order to assess virion CP composition and to dissect the CP expression strategies. Here, we show that CSDaV actually produces three subunits of the CP, which are products of different expression strategies. The major CPp21 is translated from the second start codon in the sgRNA by leaky scanning, whereas the minor CPs, p25 and p23 (renamed from p22.5), are produced by direct translation from the first start codon in the sgRNA and from *trans*‐proteolytic cleavage processing derived from the p25 precursor, respectively. Construction and further characterization of the CSDaV infectious clone represent important advances in our understanding of the CSDaV genome expression strategies, which is important for further research, such as the investigation of the role of CSDaV in CSD. CSDaV virions can now be used to inoculate citrus plants, which will allow us to study the biological activity of CSDaV on its original host, to identify the transmitting vector of CSDaV, which is so far unknown, and to evaluate a possible interaction between CSDaV and other(s) virus(es). Furthermore, as infectious cDNA clones of plant viruses have also been demonstrated to be efficient vectors for heterologous protein expression or virus‐induced gene silencing in infected plants (Gao *et al.*, 2012; Holzberg *et al.*, 2002; Kelloniemi *et al*., 2008; Lacomme *et al.*, 2003; Lindbo, 2007; Ratcliff *et al.*, 2001; Sainsbury *et al.*, 2010; Wieczorek *et al.*, 2015), the stability and efficiency of the CSDaV infectious clone also represent promising and powerful tools for citrus biotechnology.

## Results

### CSDaV virions are successfully recovered in *N. benthamiana* plants from full‐length cloned cDNA

The full‐length cDNA of CSDaV was cloned into the pJL89 binary vector under the control of the cauliflower mosaic virus (CaMV) 35S promoter to generate the 35SRbz‐CSDaV construct. After screening, we selected three clones (35SRbz‐CSDaV‐1, 35SRbz‐CSDaV‐2 and 35SRbz‐CSDaV‐3), which showed sequences identical to the original CSDaV isolate, for agroinfiltration assays. The three clones were selected as they may have different biological activities in the agroinfiltration assay. During screening, one sequenced clone showed several nucleotide mutations (U → C, G → U, G → U, C → U and G → C at nucleotide positions 4458, 5179, 5194, 5316 and 5918, respectively, in the CSDaV genome) and deletions (at nucleotide positions 5916 and 5917), which modified the CSDaV ORF structure. This latter clone was named M35SRbz‐CSDaV and was also used in the agroinfiltration experiments.

**Figure 1:**
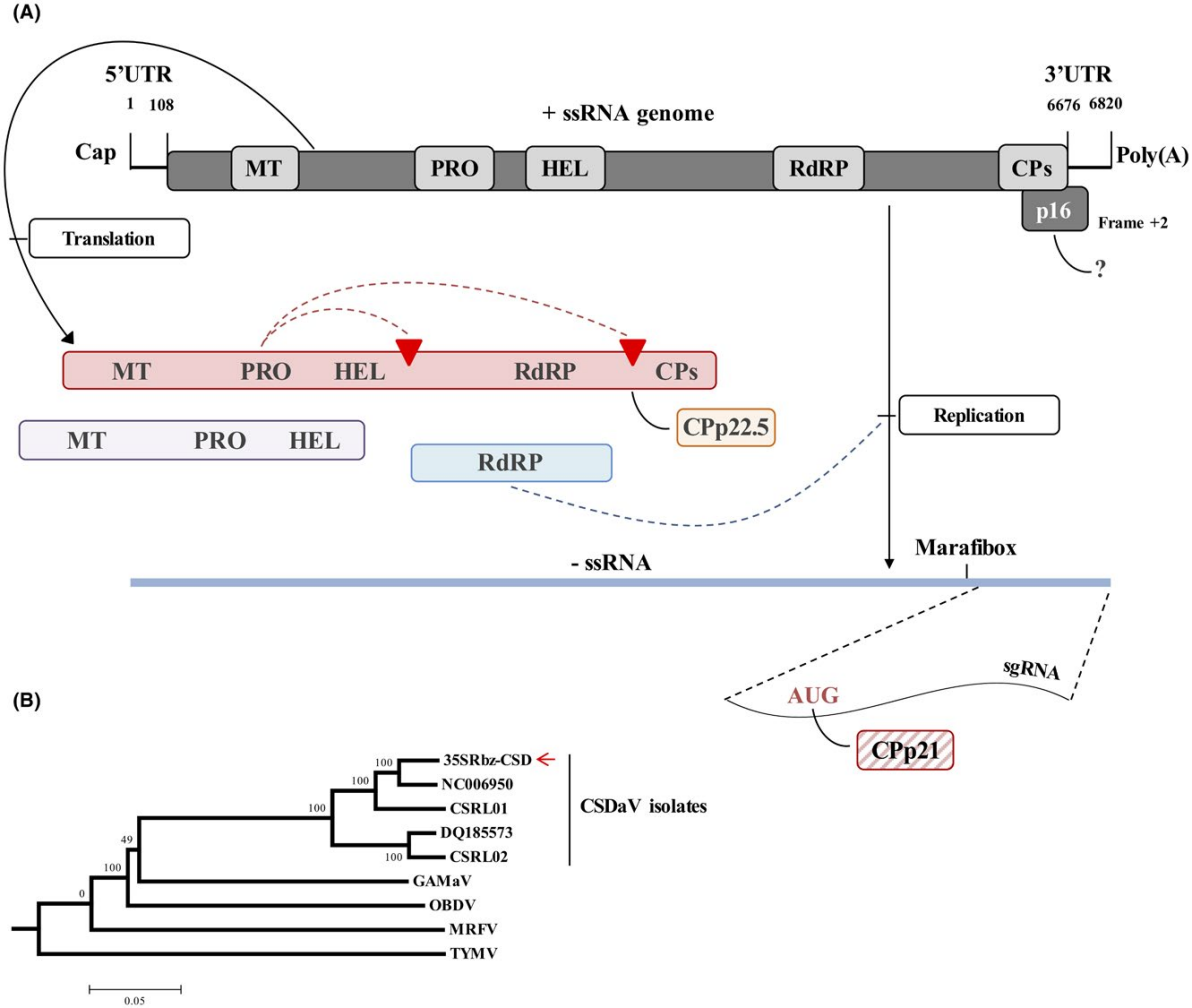
Schematic representation of a previous characterization of *Citrus sudden death‐associated virus* (CSDaV) based on its nucleotide and amino acid sequences (Maccheroni *et al*., 2005). (A) Genome organization of CSDaV (top) showing the two predicted open reading frames (ORFs, dark grey shading), with the potentially functional domains represented by grey boxes: MT, methyltransferase; PRO, protease; HEL, helicase; RdRP, RNA‐dependent RNA polymerase; CP, capsid protein and p16 (frame +2). The mechanism involved in the production of the putative p16 is still unknown and is represented by ‘?’. A polyprotein with a molecular weight of about 240 kDa (red shading), containing the functional domains for MT, PRO, HEL, RdRP and CP, is directly translated from the genomic RNA (gRNA). The predicted proteolytic cleavage sites to release CPp22.5 and RdRP from the polyprotein are indicated with red triangles. During viral replication, a complementary minus strand (blue line) is synthesized by the RdRP protein. An internal promoter (Marafibox) in the complementary minus strand triggers the transcription of a subgenomic RNA (sgRNA, indicated) from which CPp21 is directly translated (red stripes). ssRNA, single‐stranded RNA; UTR, untranslated region. (B) Phylogenetic relationships among RdRP amino acid sequences from five described CSDaV isolates, including the amino acid sequence of the 35Rbz‐CSDaV clone (indicated by a red arrow). The phylogenetic tree was reconstructed by the neighbour‐joining method. Bootstrap values are shown as percentages. *Grapevine asteroid mosaic‐associated virus* (GAMaV), *Oat blue dwarf virus* (OBDV), *Maize rayado fino virus* (MRFV) and *Turnip yellow mosaic virus* (TYMV) were included in the phylogenetic analysis as outgroup.

Analysis of the nucleotide and deduced amino acid sequences of the CSDaV clones showed characteristics consistent with those reported for other CSDaV isolates (Maccheroni *et al.*, 2005; Matsumura *et al.*, 2017). The cloned CSDaV genome has 6802 nucleotides, including 108 in the 5′‐untranslated region (5′‐UTR) and 127 in the 3’‐UTR, excluding the poly(A) tail. The highly conserved 16‐nucleotide sequence found among marafiviruses, known as the promoter (Marafibox) in the complementary minus strand for transcription of the sgRNA, was detected at nucleotide positions 5956–5971, eight to nine nucleotides upstream of the putative transcription initiation sites (CAAU and AAU, respectively) predicted by previous studies (Edwards and Weiland, 2014; Maccheroni *et al.*, 2005). The larger ORF (nucleotides 109–6675) is predicted to encode a polyprotein of 2188 amino acids and a molecular mass of about 240 kDa, which shows conserved domains for MT (nucleotides ~484–1329), PRO (nucleotides ~2803–3102), HEL (nucleotides ~3364–4053), RdRP (nucleotides ~4990–5670) and CP (nucleotides ~6082–6675) (Fig. 1A). Two putative proteolytic cleavage sites previously predicted for CSDaV (Maccheroni *et al.*, 2005) are found between HEL and RdRP (amino acid positions A^1371^/A^1372^) and between RdRP and CP (amino acid positions G^1973^/S^1974^) in the polyprotein. The previously predicted small ORF (p16) is detected between nucleotides 6260 and 6724, and putatively encodes a protein of 154 amino acids and a molecular mass of about 16 kDa (Fig. 1A). Multiple alignment and phylogenetic analyses using the full‐length sequence of the CSDaV clone and the complete genome sequences of all CSDaV isolates currently available in GenBank revealed higher sequence similarities among the CSDaV clone and isolates AY884005 (96%, accession number AY884005) and CSRL01 (93%, accession number KY110735) (Fig. 1B). The DQ185573 (accession number DQ185573) and CSRL02 (accession number KY110736) CSDaV isolates shared ≤90% sequence similarities to cloned CSDaV. The full‐length sequence of 35SRbz‐CSDaV has been deposited in the GenBank database under accession number MH784438.

**Figure 2:**
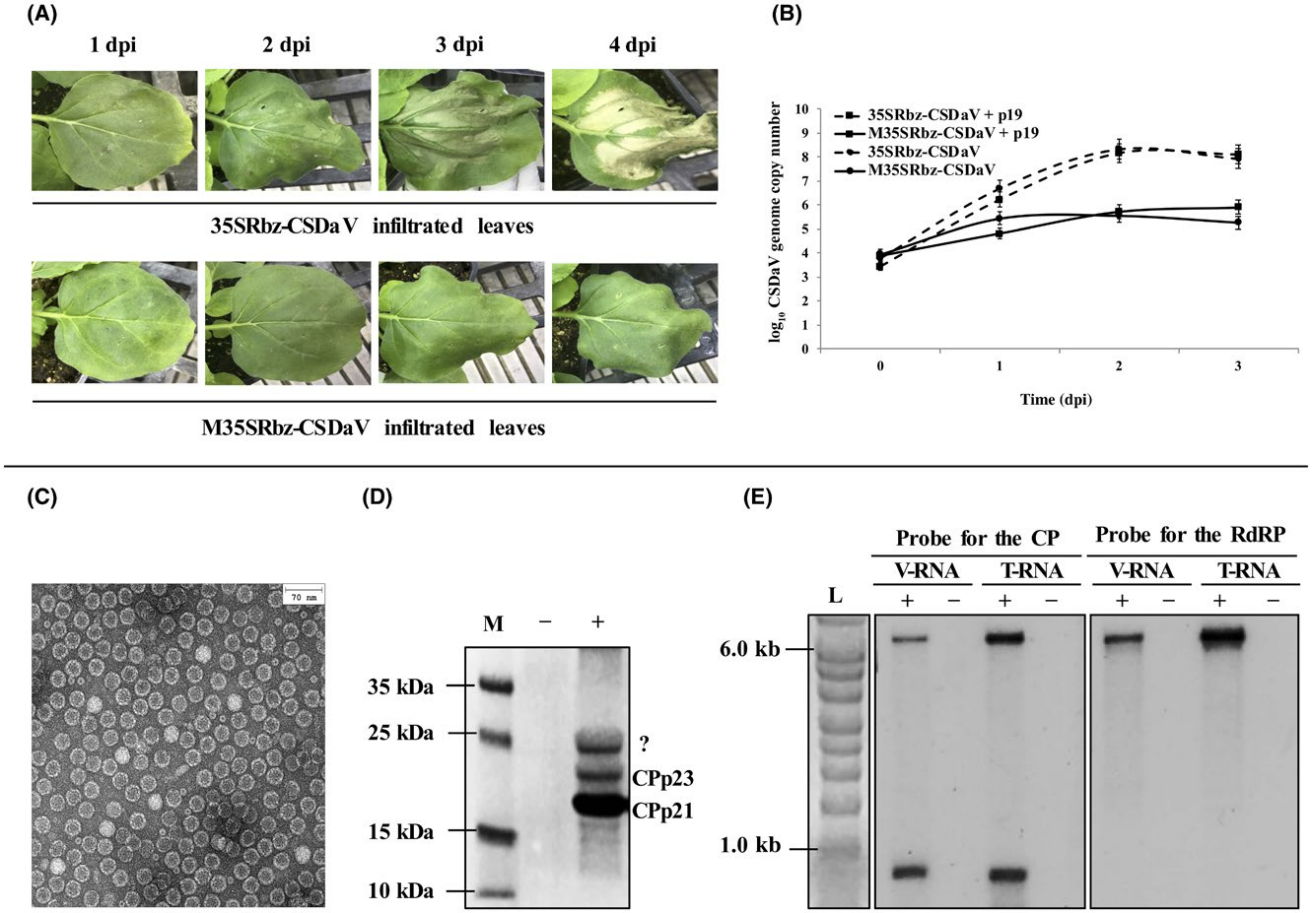
Recovery of *Citrus sudden death‐associated virus* (CSDaV) virions from full‐length cloned cDNA in *Nicotiana benthamiana* plants. (A) *Nicotiana benthamiana* leaves at 1, 2, 3 and 4 days after *Agrobacterium tumefaciens*‐mediated inoculation of the 35SRbz‐CSDaV (top) and M35SRbz‐CSDaV (bottom) clones. The 35SRbz‐CSDaV clone represents the wild‐type virus, whereas the M35SRbz‐CSDaV clone harbours multiple mutations which disturbed the CSDaV larger open reading frame (ORF). dpi, days post‐infiltration. (B) Absolute quantification of the CSDaV genome in *N. benthamiana* 35SRbz‐CSDaV‐ and M35SRbz‐CSDaV‐agroinfiltrated leaves over four time points (0, 1, 2 and 3 dpi). Quantitative polymerase chain reaction (qPCR) analysis was performed using primers to target the CSDaV capsid protein (CP) gene. Plants co‐agroinfiltrated with the respective CSDaV clone plus the pBIN‐p19 binary vector, which contains the p19 silencing suppressor gene from *Tomato bushy stunt virus *(TBSV), were included in the qPCR analysis. The plot shows the viral genome copy number (logarithmic scale, *y *axis) over the time course. Error bars are shown as the standard deviation. (C) Electron microscopy showing the isometric CSDaV virions (~ 30 nm in diameter) partially purified from *N. benthamiana* 35SRbz‐CSDaV‐agroinfiltrated leaves. Bar at the top right corresponds to 70 nm. (D) Sodium dodecyl sulfate‐polyacrylamide gel electrophoresis (SDS‐PAGE) of the partially purified virions. M, page ruler prestained ladder. (E) Northern blot analysis of the total RNA (T‐RNA) extracted from the 35SRbz‐CSDaV‐agroinfiltrated leaves and virion RNA (V‐RNA) using specific probes for the CSDaV CP (left) and RNA‐dependent RNA polymerase (RdRP, right) regions. L, RNA Millennium marker.

The infectivity of the CSDaV clones was examined in 3‐week‐old *N. benthamiana* plants by agroinfiltration assays. Clones were tested either individually or combined with the pBIN‐p19 vector containing the *Tomato bushy stunt virus* (TBSV) p19 silencing suppressor. Infiltrated leaves of all plants inoculated with 35SRbz‐CSDaV clones, either by themselves or combined with the p19 silencing suppressor, showed HR‐like necrotic symptoms on the agroinfiltrated leaves on the third day post‐infiltration (dpi), culminating with cell death on the following day (Fig. 2A). No symptoms were detected in M35SRbz‐CSDaV‐infiltrated leaves. Virus infection was then monitored by a time course reverse transcription‐quantitative polymerase chain reaction (RT‐qPCR) assay using primers to detect the CP gene of CSDaV. Total RNA from agroinfiltrated leaves was analysed at time 0 and at 1, 2 and 3 dpi. The viral copy number across these time points showed a gradual increase in 35SRbz‐CSDaV‐infiltrated leaves, being highest at 2 dpi by approximately 2.4 log units compared with time 0 (Fig. 2B). At the same time point, M35SRbz‐CSDaV‐infiltrated leaves showed an increase of about 1.5 log units (Fig. 2B). Infiltrated leaves were then collected at 2 dpi for crude virion extractions, which were analysed by transmission electron microscopy (TEM) and sodium dodecyl sulfate‐polyacrylamide gel electrophoresis (SDS‐PAGE). TEM of the negatively stained partially purified virions showed numerous isometric particles of about 30 nm in diameter (Fig. 2C). Analysis of the virion proteins by SDS‐PAGE revealed the presence of three proteins (Fig. 2D). Two of the proteins showed molecular weights of around 23 and 21 kDa, which are the expected molecular weights for the predicted CSDaV CPs (CP22.5 and CP21, respectively). However, an unexpected protein with a molecular weight of about 25 kDa was also detected and was further analysed by mass spectrometry and amino acid N‐terminal sequencing (see below). Agroinfiltrated plants were monitored during 45 dpi, but no systemic infection was detected.

### Analyses of the CSDaV RNAs confirm the transcription and encapsidation of an sgRNA

**Figure 3:**
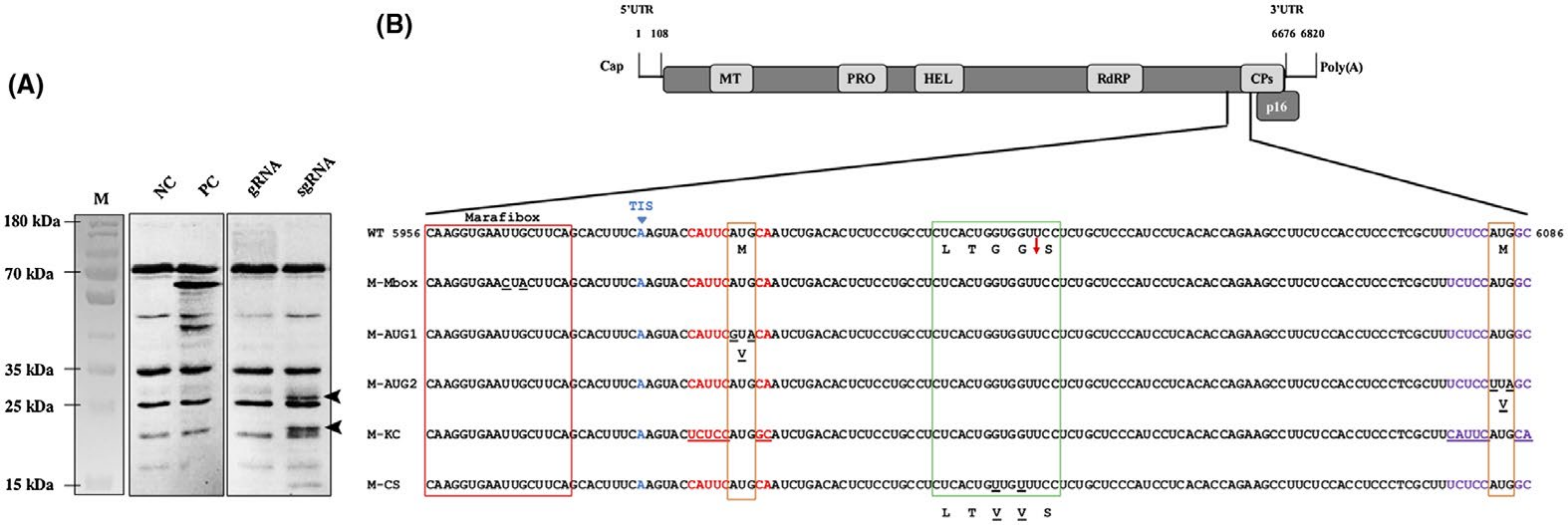
Mutant versions of the 35SRbz‐CSDaV infectious clone for analyses of *Citrus sudden death‐associated virus* (CSDaV) capsid protein (CP) expression. (A) Sodium dodecyl sulfate‐polyacrylamide gel electrophoresis (SDS‐PAGE) of the products obtained from the *in vitro* translation assay performed with genomic (g) and subgenomic (sg) transcripts *in vitro* transcribed from the 35SRbz‐CSDaV clone. NC, negative control (reactions performed without addition of the transcripts). PC, positive control (reactions performed using luciferase control RNA). M, page ruler prestained ladder. (B) Illustration of the CSDaV genome structure (top), with the targeted region enlarged to show the mutations made on the new constructs (bottom). All mutations are underlined. WT, wild‐type sequence highlighting sites putatively involved in CSDaV CP expression; M‐Mbox, mutant harbouring two nucleotide mutations in the Marafibox region; M‐AUG1, mutant harbouring two nucleotide mutations in the first initiation codon in the sgRNA; M‐AUG2, mutant harbouring two nucleotide mutations in the second initiation codon in the sgRNA; M‐KC, mutant constructed by switching the nucleotide context between the two initiation codons; M‐CS, mutant harbouring two nucleotide mutations in a region that encodes the amino acid sequence recognized by the protease for proteolytic cleavage. Red box, Marafibox core sequence in the positive‐strand RNA; orange boxes, the two initiation codons in the sgRNA; green box, proteolytic cleavage region. The exact cleavage site is indicated by a red arrow. Adenine highlighted in blue, transcription initiation site (TIS) of the sgRNA; nucleotide sequences highlighted in red, nucleotide context surrounding the first initiation codon in the sgRNA; nucleotide sequences highlighted in purple, nucleotide context surrounding the second initiation codon in the sgRNA. Important amino acids are indicated below the nucleotide sequences. MT, methyltransferase; PRO, protease; HEL, helicase; RdRP, RNA‐dependent RNA polymerase; UTR, untranslated region.

RNAs were extracted from *N. benthamiana* 35SRbz‐CSDaV‐agroinfiltrated leaves (2 dpi) and from virion extracts for northern blot analysis. Hybridization using a specific probe for the CSDaV CP region, expected to be located in the sgRNA region, showed accumulation of the gRNA (~6.8 kb in length) and an sgRNA with an expected size of about 0.8 kb in RNAs from both the agroinfiltrated leaves and virion extracts. However, hybridization using a specific probe for the CSDaV RdRP region, expected to be located outside of the sgRNA region, showed only accumulation of the gRNA (Fig. 2E). These results confirm the transcription of an sgRNA and its encapsidation in CSDaV particles. To confirm whether the 5′‐terminal sequence of the CSDaV sgRNA matches that predicted previously (Edwards and Weiland, 2014; Maccheroni *et al.*, 2005), 5′ rapid amplification of cDNA ends (5′RACE) was performed. The amplified fragments obtained from 5′RACE were cloned and sequenced. Sequencing data (data not shown) showed that the adenine at nucleotide position 5981 represents the 5′‐end of the CSDaV sgRNA and is probably the transcription initiation site (indicated as TIS in Fig. 3) for an 822‐nucleotide sgRNA [excluding the poly(A) tail].

### Further protein analyses indicate different expression strategies for the CSDaV CPs

The three virion proteins revealed by SDS‐PAGE were unexpected, as marafiviruses are believed to contain only two CPs (ICTV, 2011; Martelli *et al.*, 2002). However, we consistently observed the three proteins seen here, and each was analysed separately by tandem mass spectrometry (MS/MS). The trypsin‐treated peptide sequences derived from the proteins were found to be similar to each other, matching the predicted peptide masses of the CSDaV CPs. Considering that the three proteins have the same C‐terminal end (stop codon at amino acid position 2189), amino acid N‐terminal sequencing was performed to identify the first six N‐terminal amino acids of each protein. The sequencing results showed that the three proteins have different amino acid sequences in the N‐terminal region. The first six amino acids of the highest molecular weight (HMW) protein are MQSDTL (amino acids 1962–1967), where M is the first methionine translated from the sgRNA. The protein that showed an intermediate molecular weight (IMW) contains amino acids SSAPIL (amino acids 1974–1979) at the N‐terminus, which is located immediately after the proteolytic cleavage site predicted for CSDaV between the RdRP and CPs. The N‐terminal sequence of the lower molecular weight (LMW) protein is MASDAQ (amino acids 1992–1997), where M is the second methionine translated from the sgRNA. Considering the N‐terminal sequencing results, the molecular weights of the HMW, IMW and LMW proteins are predicted to be 25 kDa (CPp25), 23.6 kDa (CPp23) and 21.7 kDa (CPp21), respectively, which is consistent with the three proteins identified in the SDS‐PAGE gel. These results indicate that CSDaV CPs are probably produced using different expression strategies. Thus, a brief discussion is required here for a better comprehension of further experiments/results.

It is likely that the major CP (p21) is a product of direct translation of the sgRNA using the second AUG in the sgRNA (AUG_6084_) as the initiation codon (Fig. 3B). However, the CSDaV minor CPs seem to be produced by two different strategies: (1) direct translation of sgRNA, using the first AUG in the sgRNA (AUG_5994_, Fig. 3B) as the initiation codon (p25); and (2) proteolytic cleavage processing at G^1973^/S^1974 ^(p23), which could be derived from the genome‐translated polyprotein and/or from the p25 precursor translated from the sgRNA. It is also important to mention here that nucleotide comparison around the two putative initiation codons in the sgRNA (AUG_5994_ and AUG_6084_) showed that the sequence context immediately surrounding AUG_6084_ (UCUCCAUGGC) is relatively stronger than the context surrounding AUG_5994_ (CAUUCAUGCA), comparing both with the optimal translation initiation context [caA(A/C)aAUGGCg] reported previously (Joshi *et al.*, 2011; Kozak, 2002) (Fig 3B). This observation strongly indicates a leaky scanning mechanism which, although allowing the expression of the two CSDaV CPs (p25 and p21), favours the translation initiation at the start codon surrounded by a stronger context (second AUG), which is consistent with the higher abundance of the p21 protein, observed by SDS‐PAGE, compared with the p25 protein (Fig. 2D). The expression strategies suggested here for the CSDaV CPs were further evaluated by analysis, *in vivo*, of versions of the 35SRbz‐CSDaV clone harbouring mutations in sites putatively involved with CP expression (see below).

### Analyses of the 35SRbz‐CSDaV mutants reveal the expression strategies used by CSDaV for production of the CPs

To evaluate the predictions made above, independent mutant versions of the 35SRbz‐CSDaV clone were constructed by introducing mutations to disrupt the proposed CP translation initiation codons and the potential protease cleavage site, as well as to disrupt sequences putatively involved in transcription of the sgRNA. The genomic regions targeted to construct the mutants and the primer sequences used in all constructions are shown in Fig. 3B and Table S1 (see Supporting Information), respectively.

To first check whether the proposed CSDaV p25 and p21 CPs are products of direct translation from the sgRNA, genomic and subgenomic transcripts derived from the 35SRbz‐CSDaV WT clone were subjected to *in vitro* translation reactions in wheatgerm extract (WGE). SDS‐PAGE of the translated products showed that p25 and p21 CPs are probably translated from the sgRNA and not from the gRNA. Moreover, a protein with a molecular weight of around 15 kDa was also translated from the sgRNA transcripts, which could indicate that the putative p16 protein encoded by the 3′‐proximal ORF could also be translated from the sgRNA (Fig. 3A).

**Figure 4:**
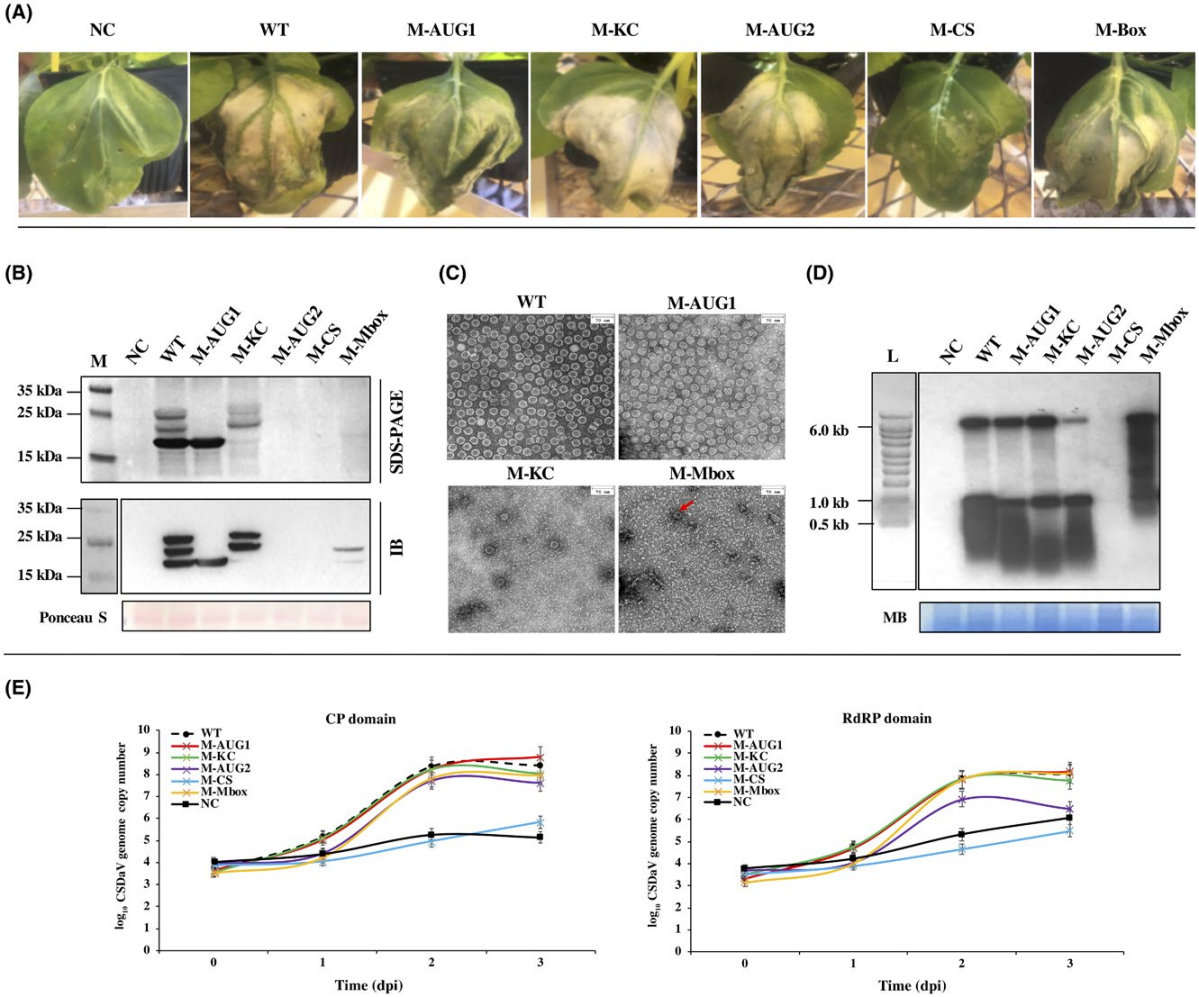
Biological activity of the 35SRbz‐CSDaV mutants in *Nicotiana benthamiana* plants. (A) Symptoms developed in *N. benthamiana* leaves in response to agroinfiltration of the independent mutants. NC, negative control (M35SRbz‐CSDaV); WT, wild‐type infectious clone (35SRbz‐CSDaV). (B) Comparison of capsid protein (CP) production among the mutants. Virion proteins (top) and total proteins (bottom) were analysed by sodium dodecyl sulfate‐polyacrylamide gel electrophoresis (SDS‐PAGE) (top) and immunoblotting (IB) using the anti‐CSDaV‐CP antibody (bottom). The Ponceau S‐stained ribulose‐1,5‐bisphosphate carboxylase/oxygenase large subunit was used as a loading control for the total proteins. M, page ruler prestained ladder. (C) *Citrus sudden death‐associated virus* (CSDaV) mutants that showed any CP production had their partially purified virions analysed by transmission electron microscopy (bars at the top right correspond to 70 nm). (D) Comparison of RNA accumulation among the mutant versions of 35SRbz‐CSDaV by northern blot of the total RNA using specific probes for the CSDaV CP region. The methylene blue (MB)‐stained blot was used as a loading control of total RNA. L, RNA Millennium marker. (E) Absolute quantification of the M‐Mbox, M‐AUG1, M‐AUG2, M‐KC and M‐CS genomes in *N. benthamiana*‐agroinfiltrated leaves over four time points [0, 1, 2 and 3 days post‐infiltration (dpi)]. Quantitative polymerase chain reaction (qPCR) analysis was performed using primers to target the CSDaV CP and RNA‐dependent RNA polymerase (RdRP) genes. The plot shows the viral genome copy number (logarithmic scale, *y *axis) over the time course. Error bars are shown as the standard deviation.

The biological activity of the CSDaV mutant constructs was tested *in vivo* by agroinfiltration in *N. benthamiana* plants. Plants were monitored for HR‐like symptoms and different assays were performed in order to check the ability of the CSDaV mutants for replication and virion assembly. To confirm the role of the Marafibox in the transcription of the sgRNA, nucleotide substitutions were made in the Marafibox core sequence at positions 5965 (U → C) and 5967 (G → A) to obtain the mutant M‐Mbox (Fig. 3B). Plants agroinfiltrated with M‐Mbox showed identical WT‐like symptoms (Fig. 4A). However, northern blot analysis (Fig. 4D) and absolute quantification of CSDaV gRNA by RT‐qPCR (Fig. 4E) showed that the two nucleotide mutations in Marafibox reduced the transcription of the sgRNA by approximately 1 log unit compared with the sgRNA accumulation of the WT infectious clone. The accumulation of gRNA was not affected by these mutations (Fig. 4D,E); however, the production of CPs was affected by the reduction in sgRNA transcription (Fig. 4B). Two days after inoculation, the amount of CPp21 was much lower than the WT level and CPp25 was reduced to an undetectable level (Fig. 4B). Interestingly, the production of CPp23 was also strongly reduced (Fig. 4B). If CPp23 was derived via processing from the full polyprotein, it would not be expected to be reduced here, as gRNA was not reduced. Thus, unlike previous predictions (Maccheroni *et al.*, 2005), this suggests that CPp23 is more likely to be a product of proteolytic cleavage processing derived or partially derived from a precursor translated from the sgRNA. Virion purification from the M‐Mbox‐agroinfiltrated leaves was performed at 2 dpi, but only a few particles were found by TEM (Fig. 4C).

Mutants M‐AUG1 and M‐AUG2 were constructed by introducing nucleotide mutations to disrupt the proposed initiation codons of the minor p25 and major p21 CPs (AUG_5994_ and AUG_6084_, respectively) (Fig. 3B). Disruption of the first AUG_5994_ (M‐AUG1) completely eliminated the production of both proposed forms of minor CPs (p25 and p23) (Fig. 4B). As for the M‐Mbox results above, because gRNA replication is still high, this also suggests that CPp23 is not derived from translation of the gRNA and polyprotein processing, but further indicates that p23 is likely to be a proteolytic cleavage product of the p25 precursor translated from the sgRNA. The accumulation of CPp21, symptoms of M‐AUG1‐agroinfiltrated leaves, viral RNA accumulation (both gRNA and sgRNA), and amount and structure of assembled virions all showed similar levels to the WT infectious clone (Fig. 4A–E). By contrast, although plants agroinoculated with M‐AUG2 showed similar WT HR‐like symptoms (Fig. 4A), the disruption of the second AUG_6084_, in addition to the knockdown of CPp21, also affected the production of the p25 and p23 CPs (Fig. 4B), which completely abolished virion assembly, suggesting that p21 is essential for virion assembly. Whether or not it is related to the absence of CPs, the accumulation of the gRNA from the M‐AUG2 mutant was reduced by approximately 1.3 log units compared with WT (Fig. 4D,E), but sgRNA accumulated at the WT level.

To confirm whether the translation of p25 and p21 CPs from the initiation sites at positions AUG_5994_ and AUG_6084_, respectively, follows the leaky scanning mechanism, mutant M‐KC was constructed by switching the nucleotide contexts between the two AUGs. Contrary to the WT, the new mutant had a strong nucleotide context surrounding the first AUG and a weak context surrounding the second AUG (Fig. 3B). M‐KC‐agroinfiltrated plants accumulated WT levels of gRNA and sgRNA, and also showed similar WT‐like symptoms (Fig. 4A,D,E). The production of p25 and p23 CPs was clearly detected, but p21 production was greatly reduced (Fig. 4B), which confirms that the nucleotide contexts surrounding the two AUGs are critical for the translation strategy used by CSDaV, which seems to favour the translation of CPp21. By TEM, the number of virions obtained from M‐KC‐agroinfiltrated leaves at 2 dpi was much less than that for the WT infectious clone (Fig. 4C). Furthermore, many particles not completely assembled were found, suggesting that a larger amount of p21 is necessary to maintain virion assembly at WT levels. Mutant M‐CS was constructed to assess the importance of the proposed cleavage site between RdRP and the CPs at amino acid positions G^1973^/S^1974^. The mutations involved two amino acid substitutions (GlyGly^1973^ → ValVal^1973^) (Figs 3B, 4D,E). No symptoms and no accumulation of viral RNAs or CPs were detected, indicating that the G^1973^/S^1974 ^cleavage site is essential for viral replication, most probably as it releases the viral RdRP from the polyprotein (Fig. 4A,B,D,E).

### Complementation assay of CP‐deficient mutants shows that CPp23 is released from the p25 precursor by *trans*‐protease activity

**Figure 5:**
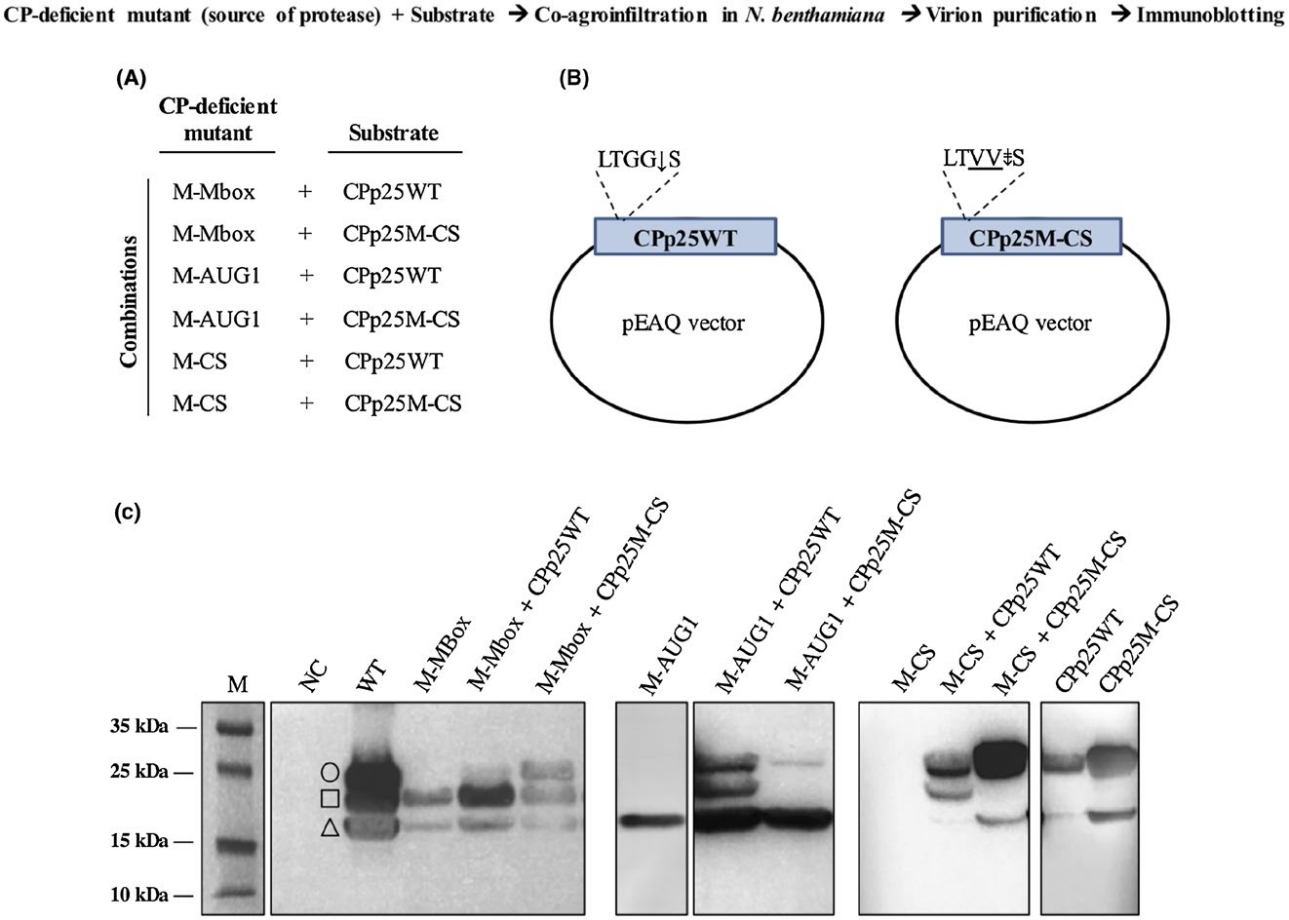
*In vivo* *trans*‐protease activity assays were performed by co‐agroinfiltration of capsid protein (CP)‐deficient mutants (M‐Mbox, M‐AUG1 and M‐CS), as sources of *Citrus sudden death‐associated virus* (CSDaV) protease, with the putative substrates in *Nicotiana benthamiana* plants. (A) Combinations of CP‐deficient mutants and substrates used in the co‐agroinfiltration assay. (B) Illustration of the two substrates used in this assay: CPp25WT (p25 wild‐type CP individually cloned into the pEAQ vector) and CPp25M‐CS (p25 harbouring a disrupted proteolytic cleavage site individually cloned into the pEAQ vector). (C) Virion purification was performed with the co‐infiltrated leaves and analysed by immunoblotting using anti‐CSDaV‐CP antibody. The M‐Mbox, M‐AUG1 and M‐CS mutants, as well as the CPp25WT and CPp25M‐CS clones, were individually analysed as controls. The full‐length CPp25 substrate from the wild‐type (WT) clone is indicated with a circle. The CPp23 cleaved product is indicated with a square. CPp21 is indicated with a triangle. M, page ruler prestained ladder.

Taken together, the *in vivo* analysis of the 35SRbz‐CSDaV mutants suggests that the CSDaV CPp23 is most likely a proteolytic cleavage product derived from a larger precursor (CPp25) which results from translation of the sgRNA. This suggests that the CSDaV protease has both *cis* and *trans* proteolytic activities. The *cis*‐protease activity must occur in order to process the polyprotein, and the *trans*‐protease activity must be responsible for releasing CPp23 from the CPp25 precursor. To test this, a CP complementation assay was performed by co‐infiltration of *N. benthamiana* plants with the CP‐deficient mutants (M‐Mbox, M‐AUG1 and M‐CS, see Figs 3 and 4) as sources of the CSDaV protease, and CPp25WT and CPp25M‐CS, individually cloned into the pEAQ vector (Fig. 5A,B), as substrates. As shown above, the M‐Mbox mutant produces small amounts of CPs p23 and p21 when it is agroinfiltrated in *N. benthamiana* plants (Fig. 4B). The low production of CPp21 reflects the low accumulation of sgRNA by this mutant, as this CP subunit is directly translated from the sgRNA. However, no detectable accumulation of CPp25, but accumulation of CPp23, might indicate two processes: (1) all the translated CPp25 from sgRNA is proteolytic cleaved to release CPp23; or (2) the translational machinery favours CPp21 translation by leaky scanning and CPp23 is cleaved from the polyprotein (although the above results suggest that this is unlikely).

Compared with the expression profile of the M‐Mbox mutant, co‐agroinfiltration with the combination M‐Mbox plus CPp25WT showed the production of similar amounts of CPp21, but greater accumulation of CPp23 (Fig. 5C) and very low accumulation of CPp25. Because CPp25 results from translation of CPp25WT, this suggests that CPp25 is probably preferentially used as a precursor and is processed by *trans*‐proteolytic cleavage (the protease derived from the M‐Mbox mutant) to release CPp23. Consistently, when mutant M‐Mbox was co‐infiltrated with CPp25M‐CS, which has the proteolytic cleavage site disrupted by two amino acid mutations, production of CPp25 was restored and accumulation of CPp23 was detected at a similar level to that of the M‐Mbox mutant alone (Fig. 5C). Co‐infiltration of M‐AUG1, a CPp25‐ and CPp23‐deficient mutant, with CPp25WT was able to restore the production of CPp25 and CPp23, whereas co‐infiltration with M‐AUG1 and CPp25M‐CS restored only the production of CPp25 (Fig. 5C). In order to check whether the *trans*‐proteolytic cleavage processing of CPp25 is carried out by the virus protease and not by a plant protease, which could be induced by viral infection, we co‐infiltrated the M‐CS mutant, which does not replicate or produce any CP proteins because of two amino acid mutations at the cleavage site that release RdRP from the polyprotein, with either CPp25WT or CPp25M‐CS. Co‐infiltration of M‐CS and CPp25WT showed the production of CPp25, CPp23 and a very small amount of CPp21 (Fig. 5C). However, co‐infiltration of the M‐CS mutant and CPp25M‐CS resulted in the production of only p25 and p21 CPs (Fig. 5C). Individual agroinfiltration of CPp25WT and CPp25M‐CS was also performed as a control. Both showed the expression of p25 and p21 CPs, but no cleaved product (CPp23) was detected. Taken together, these results, contrary to previous predictions (Maccheroni *et al.*, 2005), strongly suggest that CSDaV CPp23 is proteolytically cleaved from the p25 precursor by viral *trans*‐protease activity, instead of being derived from the polyprotein by *cis*‐protease activity.

## Discussion

CSDaV has been shown to be the virus most associated with CSD disease in Brazil (Maccheroni *et al.*, 2005; Matsumura *et al.*, 2017). However, the low viral titre and the occurrence of mixed viral infections in CSD‐affected plants (Matsumura *et al.*, 2017) have hampered virion purification and transmission experiments of CSDaV, which have delayed studies on the role of this virus in CSD and even on its molecular biology. In this work, we were able to efficiently recover CSDaV virions by constructing and agroinoculating a full‐length cDNA infectious clone of CSDaV in *N. benthamiana* plants. Although agroinoculated plants showed a very severe local HR‐like response to CSDaV infection, we were able to purify large amounts of virions from the agroinfiltrated leaves just before the HR symptoms appeared (only 2 dpi). This allowed us to biologically characterize CSDaV for the first time, as most of the information on this virus is based only on predictions from the available nucleotide/amino acid sequences (Maccheroni *et al.*, 2005; Matsumura *et al.*, 2017).

The high replication rate of CSDaV derived from our infectious clone in *N. benthamiana*‐agroinfiltrated leaves was crucial to clearly detect/differentiate the gRNA and sgRNA of CSDaV. Consistent with previous predictions for CSDaV and other marafiviruses (Maccheroni *et al.*, 2005; Martelli *et al.*, 2002), we demonstrated that CSDaV sgRNA is synthesized from an internal promoter (known as Marafibox) in the complementary minus strand RNA. We also identified the adenine nucleotide, located 10 nucleotides downstream from the 3′‐end of the Marafibox core sequence, as the 5′‐end nucleotide of the sgRNA. This adenine nucleotide, nine to ten nucleotides downstream from the Marafibox, is very conserved among the known marafiviruses, and is also the 5′‐end nucleotide of the *Oat blue dwarf virus* (OBDV) sgRNA, which is also a member of the genus *Marafivirus* (Edwards and Weinland, 2014). This may suggest that marafiviruses share the same initiation site in the transcription of the sgRNA.

**Figure 6:**
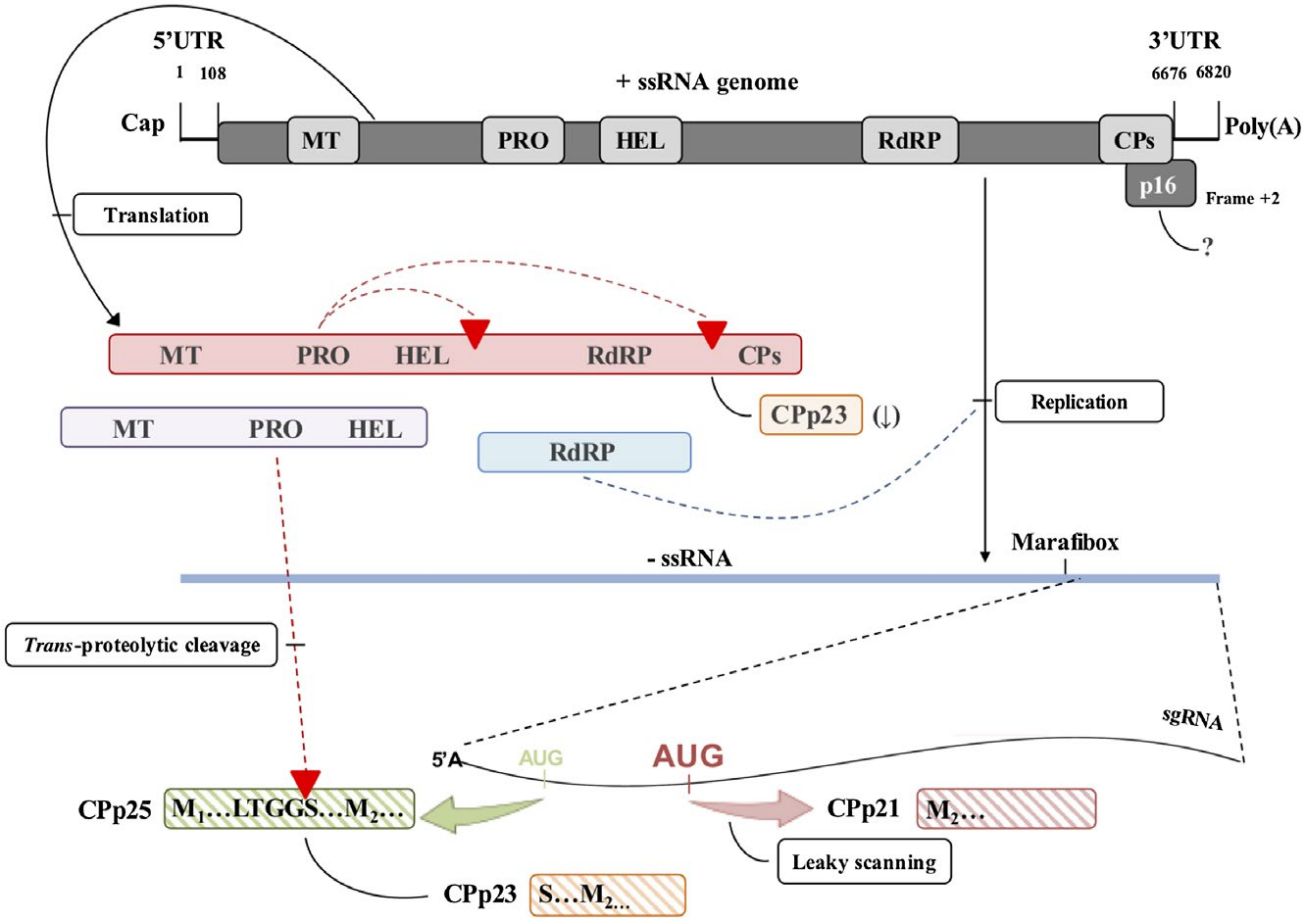
The expression strategies used by *Citrus sudden death‐associated virus* (CSDaV) for capsid protein (CP) production: an update of Fig. 1 based on the results from the present work. From the top: genome organization of CSDaV showing the positive‐sense, single‐stranded (ss) genomic RNA (gRNA) and the two predicted open reading frames (ORFs, dark grey shading), where the potentially functional domains are represented by grey boxes: MT, methyltransferase; PRO, protease; HEL, helicase; RdRP, RNA‐dependent RNA polymerase; CP, capsid protein and p16 (frame +2). The mechanism involved in the production of the putative p16 is still unknown and is represented by ‘?’. A polyprotein with a molecular weight of about 240 kDa (red shading), containing the functional domains for MT, PRO, HEL, RdRP and CP, is directly translated from the gRNA. *Cis*‐proteolytic cleavage is predicted to occur in two cleavage sites (indicated by red triangles) to release RdRP from the polyprotein. This process must also release a small amount of CPp23, indicated by ‘–’. During the replication process of the viral genome, a complementary minus strand (blue line) is synthesized by the RdRP protein. An internal promoter (Marafibox) in the complementary minus strand triggers the transcription of a subgenomic RNA (sgRNA, indicated) from which the three CP subunits are derived. The major CPp21 (red stripes) is translated from the second start codon, favoured by a leaky scanning mechanism. The minor CPp25 (green stripes) and CPp23 (orange stripes) are produced by direct translation from the first start codon in the sgRNA and by *trans*‐proteolytic cleavage processing (red triangle) derived from the p25 precursor, respectively. UTR, untranslated region.

The characterization of the CSDaV sgRNA in this work was essential to better understand the biological significance of its transcription. It is very well known that the synthesis of sgRNAs is a common strategy used by positive‐sense RNA viruses for the differential expression of specific viral genes, normally 3′‐proximal genes (Koev and Miller, 2000; Sztuba‐Solinska *et al.*, 2011). Transcription of sgRNA by marafiviruses, including CSDaV, has been reported as a strategy for the expression of the major CP exclusively, whereas the minor CP has been suggested to be proteolytically cleaved from the polyprotein translated from the gRNA (Maccheroni *et al.*, 2005; Martelli *et al.*, 2002). In this work, we have shown that, different from previous reports for other known marafiviruses, CSDaV actually produces three sgRNA‐derived forms of the CP, each of which is a virion component. These are the major CPp21 and two minor CPs (CPp23 and CPp25). *In vivo* analyses of mutant versions of the CSDaV infectious clone helped us to uncover the expression strategies used by CSDaV for the production of these three forms of CP. We confirmed that the major CPp21 is produced by direct translation from the second initiation codon (AUG_6084_) in the sgRNA, as predicted previously by Maccheroni *et al. *(2005). In addition, we were able to biologically demonstrate that the expression of p21 protein is strongly influenced by the nucleotide contexts surrounding the two initiation codons in the sgRNA (AUG_5994_ and AUG_6084_), showing that the major CSDaV CP uses the ribosome leaky scanning mechanism to be translated. As far as we know, the leaky scanning mechanism has never been reported for any virus from the family *Tymoviridae*, suggesting that the direct translation of two in‐frame proteins from the same sgRNA might be an evolutionary strategy used by CSDaV to allow the higher production of proteins required for late functions, such as virion assembly and/or infectivity. Direct translation from the first initiation codon (AUG_5994_) in the sgRNA gives rise to one of the two minor CPs: CPp25. Our results also strongly suggest that CPp25 is the precursor that is processed by *trans*‐proteolytic cleavage to release the other minor CSDaV CP: CPp23. Thus, unlike other marafiviruses and contrary to previous predictions for CSDaV, we show that the minor CSDaV, CPp23, is also derived from sgRNA. However, we also show that the proteolytic cleavage of the polyprotein at the G^1973^/S^1974 ^cleavage site is essential for CSDaV replication, which indicates that CPp23 must also be cleaved from the polyprotein. Nevertheless, it is likely that CPp25 (from sgRNA) accumulates to high levels in infected cells, compared with the accumulation of the large polyprotein (from the gRNA), and thus the contribution of the proteolytic cleavage of the polyprotein to the production of CPp23 is likely to be minimal. In a previous study, Edwards and Weinland (2014) reported that the OBDV minor CP is cleaved from both the polyprotein and the precursor translated from the sgRNA; although the mechanisms involved were not clearly elucidated, they suggested that it might be possible that OBDV is in an evolutionary transition towards CP production only through sgRNAs. This is valuable if we consider that the production of minor CPs is involved in the maintenance of stoichiometry between minor and major viral CPs. As shown here, the major CPp21 is produced in large amounts from CSDaV sgRNA; thus, CSDaV might require expression strategies to also produce larger amounts of minor CPs in order to provide the stoichiometric balance needed for efficient virion assembly. All the expression strategies used by CSDaV to produce CPs are summarized in Fig. 6.

Undoubtedly, the results obtained here significantly improve our knowledge on the molecular biology of marafiviruses in general and, in particular, of CSDaV. The fact that we could quickly recover CSDaV virions in *N. benthamiana *plants from cloned cDNA greatly facilitated our studies. However, this plant model does not allow CSDaV systemic infection, which could restrict further studies, such as the functional analysis of a putative movement protein. Thus, we are now aiming to evaluate the biological activity of CSDaV on citrus. Being able to systemically infect citrus plants with CSDaV virions represents an opportunity to overcome the challenges faced by previous studies during the investigation of the association between CSDaV and CSD‐affected plants. Furthermore, the identification of a possible CSDaV vector and possible interactions between CSDaV and other viruses, such as *Citrus tristeza* *virus* (CTV), for example, which is always found in co‐infections with CSDaV in field plants (Matsumura *et al.*, 2017), are other examples of future investigations. Finally, the suitable characteristics found in the CSDaV infectious clone, such as the small size, fast replication and easy manipulation, make this infectious clone a good candidate for application in citrus biotechnology.

## Experimental Procedures

### Virus source and RNA extraction

CSDaV‐infected citrus plants, previously collected in a grove located in the municipality of Comendador Gomes (southwestern Minas Gerais State), Brazil (Matsumura *et al.*, 2016), were used as viral sources in this work. The total RNA was extracted from the petiole tissue of the leaves following the cetyltrimethylammonium bromide (CTAB)‐based protocol (Bekesiova *et al*., 1999).

### Construction of a full‐length cDNA clone of CSDaV

The full‐length cDNA clone of CSDaV was constructed using the In‐Fusion cloning system (Clontech, Mountain View, CA, USA). A set of primers (Table S1) was designed to insert two fragments, spanning the full CSDaV gRNA, into the pJL89 vector (Lindbo, 2007) (Fig. S1, see Supporting Information). The fragments were inserted immediately downstream of the double‐enhanced CaMV 35S promoter (2 × 35S) and upstream of the hepatitis delta virus ribozyme (HDV‐Rbz) by 20‐base complementary overlapping ends.

The cDNA was synthesized from 500 ng of total RNA obtained from the CSDaV‐infected tissues using Superscript™ III reverse transcriptase (Invitrogen, Carlsbad, CA, USA) and oligo(dT) primer, according to the manufacturer’s instructions. The linearized pJL89 vector (4675 bp) and the CSDaV fragments were obtained by PCR with their respective primers, using CloneAmp HiFi PCR premix (Clontech), following the manufacturer’s protocol. The amplicons were gel purified using a Zymoclean™ Gel DNA Recovery Kit (Zymo Research Corporation, Irvine, CA, USA) (Fig. S1). The In‐Fusion reactions were performed using 100 ng of the linearized vector, 100 ng of the respective purified fragment, 1 × In‐Fusion HD Enzyme Premix (Clontech) and deionized water to 10 µL of total volume. The reactions were incubated at 50 °C for 15 min, and then placed on ice for transformation using *Escherichia coli* Stellar™ competent cells (Clontech). The transformants were screened by colony PCR using CSDaV‐specific primers (Table S1). The PCR‐positive colonies were selected for plasmid purification using a Maxiprep Purification Kit (Qiagen, Valencia, CA, USA). The full‐length cDNA clones of CSDaV were confirmed by digestion with *Rsr*II restriction enzyme (NEB, Ipswich, MA, USA) and Sanger sequencing using primers designed along the CSDaV genome (Fig. S1 and Table S1). Sequences were analysed using Contig Express (Vector NTI, Invitrogen, Waltham, MA, USA) and the ORFs were confirmed using the ORF finder function of the SnapGene software version 3.3 (http://www.snapgene.com/).

### Agroinfiltration assay

The selected CSDaV clones were introduced into *A. tumefaciens* strain GV3101 by electroporation using a Gene Pulser apparatus (Bio‐Rad, Richmond, CA, USA) according to the manufacturer's specifications. Pre‐inoculum cultures of the selected clones were grown overnight at 28 °C in Luria–Bertani (LB) medium containing 50 μg/mL of kanamycin. An aliquot of the pre‐inoculum was inoculated in 25 mL of L‐MESA medium (LB medium supplemented with 10 mm 2‐(N‐morpholino) ethanesulfonic acid [MES] and 20 μm acetosyringone), containing the same antibiotic, and grown overnight at 28 °C to an optical density at 600 nm (OD_600 nm_) of between 0.8 and 1.2. The cells were centrifuged for 10 min at 6000 *g*, resuspended in agroinduction medium (10 mm MgCl_2_, 100 µm acetosyringone and 10 mm MES, pH 5.7) to an OD_600 nm_ of 0.8 and incubated at room temperature (RT) for 5 h in the dark. The same procedure was performed for the *A. tumefaciens* colony transformed with the pBIN‐p19 binary vector, which contains the p19 silencing suppressor gene from TBSV. Cultures of *A. tumefaciens* containing the CSDaV constructions were infiltrated alone or combined with cultures containing the p19 silencing suppressor (1 : 1) on the abaxial surface of young expanded leaves of *N. benthamiana* plants. We used three plants for each treatment [35SRbz‐CSDaV, M35SRbz‐CSDaV (clone showing several mutations), 35SRbz‐CSDaV + pBIN‐p19 and M35SRbz‐CSDaV + pBIN‐p19] and infiltrated four leaves per plant. A plant infiltrated with *A. tumefaciens* containing only the p19 silencing suppressor was used as a negative control. The agroinfiltrated plants were maintained in a glasshouse under constant conditions and monitored for symptom development.

### Real‐time RT‐qPCR

Viral accumulation was checked at 0, 1, 2 and 3 dpi. Total RNAs were extracted from agroinfiltrated leaves using Trizol reagent (Life Technologies, Carlsbad, CA, USA), treated with RQ1 RNase‐free DNase I (Promega, Madison, WI, USA), following the manufacturer’s instructions, and purified by phenol–chloroform extraction. The purified RNAs were reverse transcribed using a High‐Capacity cDNA Reverse Transcription Kit (Applied Biosystems, Carlsbad, CA, USA), following the manufacturer’s protocol. qPCR was performed to detect the CP gene of CSDaV using 5 μL of SsoAdvanced Universal SYBR Green Supermix (Bio‐Rad, Foster City, CA, USA), 300 nm of each primer (CSDaV‐CPF/CSDaV‐CPR), 1 μL of the 1 : 5 diluted cDNA and water up to a total volume of 10 μL. The thermocycling conditions were as follows: 95 °C for 3 min, followed by 40 cycles of 95 °C for 10 s and 55 °C for 30 s. The primer sequences are listed in Table S1.

The same procedure was performed with samples from serial dilutions of plasmid 35SRbz‐CSDaV in order to establish the standard curve. The plasmid copy number was calculated as described by Plumet and Gerlier (2005). The standard curve obtained (not shown) was then used to estimate the CSDaV viral copy number in the tested samples. RT‐qPCR experiments were performed in three biological replicates and three technical repeats.

### Virion purification, SDS‐PAGE and TEM

CSDaV virions were purified according to Burgyan and Russo (1998). Infected leaves were collected at 2 dpi and homogenized with 5 mL/g of 0.2 m sodium acetate, pH 5.0. The obtained solution was filtered through cheesecloth and left overnight at 4 °C. The solution was clarified by low‐speed centrifugation (8000 *g* for 10 min at 4 °C) and the virions in the supernatant were precipitated with 8% (w/v) polyethylene glycol (PEG8000) and 1% (w/v) NaCl, followed by a low‐speed centrifugation (8000 *g* for 10 min at 4 °C). The pellet was resuspended in 10 mm Tris‐HCl buffer, pH 7.8. The virion extracts were analysed by SDS‐PAGE using 12.5% polyacrylamide gel, followed by Coomassie Brilliant Blue R‐250 (Fisher Biotech, Fair Lawn, NJ, USA) staining (Sambrook and Russell, 1989).

The virion extracts (5 μL of 1 : 10 diluted extracts) were loaded onto Formvar‐carbon‐coated grids, left for 2 min and drained with filter paper. The grids were stained using five drops of 1% uranyl acetate, drained and air dried. The stained grids were examined with a JEOL 2100F transmission electron microscope (Peabody, MA, USA) at an accelerating voltage of 200 kV.

### Northern blot and 5′RACE

Total RNAs extracted from *N. benthamiana* 35SRbz‐CSDaV‐agroinfiltrated leaves and from the virion extracts were denatured with glyoxal at 55 °C for 30 min, electrophoresed on 1% agarose gels and transferred to Hybond‐NX membrane (GE Amersham, Piscataway, NJ, USA) by capillary transfer. RNAs were fixed to the membrane by cross‐linking in UV light and the blots were stained with methylene blue. The membranes were hybridized with ^32^P‐labelled probes to detect the positive‐strand RNA. To make the probes, amplified and gel‐purified DNA fragments covering nucleotides 6229–6590 (CP) and 5342–5708 (RdRP), and containing the T7 promoter, were employed as templates for *in vitro* transcription reaction using T7 RNA polymerase (Ambion MAXIscript T7 Transcription Kit, Vilnius, Lithuania) in the presence of [α‐^32^P]UTP, according to the manufacturer’s instructions. After hybridization, the membranes were washed once in 2 × SSC (1 × SSC is 0.15 m NaCl plus 0.015 m sodium citrate) and 0.1% SDS at RT for 15 min, once in 0.5 × SSC/0.1% SDS at RT for 15 min and once with 0.1 × SSC/0.1% SDS at 65 °C for 15 min. The membranes were exposed to Premium X‐Ray film (Phenix Research Products) for hybridization detection.

To determine the 5′‐end of the CSDaV sgRNA, virion RNA was used as template for the synthesis of the cDNA employing a gene‐specific antisense primer (GSP1), designed to anneal nucleotides downstream from the putative 5′‐end of the sgRNA (Table S1). The cDNA was synthesized using SuperScript II (Thermo Fisher Scientific, Waltham, MA, USA), following the manufacturer’s protocol. RNA template was removed by the addition of 1 µL of RNAse mix (Thermo Fisher Scientific), followed by incubation at 37 °C for 30 min. The reaction was purified using the DNA Clean & Concentrator −5 Kit (Zymo Research Corporation), following the manufacturer’s instructions, and the cDNA was tailed with dCTP following the instructions from the 5′RACE System for Rapid Amplification of cDNA Ends Kit (Thermo Fisher Scientific). The dC‐tailed cDNA was then amplified by one or two rounds of nested PCRs using the abridged anchor forward primer, designed to bind the dC‐tailed region, and GSP2 or GPS3 reverse primers (Table S1). The amplified products were gel purified and cloned into pCR‐Blunt II‐TOPO vector (Thermo Fisher Scientific) following the manufacturer’s protocol. We randomly selected 30 colonies for plasmid purification and Sanger sequencing. The sequences obtained were aligned in ClustalX (Larkin *et al*., 2007) and the most predominant 5′‐end sequence was considered to be the 5′‐end of the CSDaV sgRNA.

### Mass spectrometry and N‐terminal sequencing

Purified virions were electrophoresed in two SDS‐PAGE gels as described above. One of the gels was subjected to Coomassie Brilliant Blue staining and the protein bands were excised for mass spectrometry analysis. Mass spectrometry was performed as described by Pope *et al. *(2004) at the University of California, Davis Proteomics Core (UCDPC, http://proteomics.ucdavis.edu) by liquid chromatography‐tandem mass spectrometry (LC‐MS/MS) (Q‐Exactive, Thermo Scientific, Waltham, MA, USA). Samples were analysed using X! Tandem version Alanine 2017.2.1.4 [GPM, The Global Proteome Machine initiative, http://thegpm.org (accessed on 8 July 2018)] set up to search the CSDaV database assuming digestion with trypsin. The MS/MS data were analysed using Scaffold4 [version Scaffold_4.8.3, Proteome Software Inc., Portland, OR, USA; http://www.proteomesoftware.com/products/scaffold/ (accessed on 8 July 2018)]. The proteins from the other gel were transferred to polyvinylidene difluoride (PVDF; Bio‐Rad, USA) membrane by electroblotting at 70 V for 40 min. The membrane was stained with Coomassie Brilliant Blue, destained with 50% methanol and subjected to N‐terminal amino acid sequencing by automated Edman degradation on an ABI Procise 494‐HT (Applied Biosystems, Foster City, CA, USA) sequencer at UCDPC.

### Construction of the 35SRbz‐CSDaV mutants and *in vitro* and *in vivo* analyses

The 35SRbz‐CSDaV plasmid was employed as template to generate a set of independent mutants (Fig. 3B) using two partially complementary overlapping mutagenic primers (Table S1). Reverse PCR amplification was performed in two rounds using CloneAmp HiFi PCR premix (Clontech). The first round was performed by adding the forward and reverse primers in two independent reactions, and consisted of three cycles of 98 °C for 10 s, the respective annealing temperature of the primer for 15 s and 72 °C for 1 min. The reactions with the forward and reverse primers were mixed and then subjected to a second round of 16 cycles of amplification following the same conditions. The amplicons were gel purified and approximately 100 ng of the mutated linearized plasmids were re‐ligated using the In‐Fusion system, as described previously. The re‐ligated plasmids were transformed into *E. coli* (DH5α) and the transformants were screened by colony PCR using CSDaV‐specific primers. Plasmids were purified using a QIAprep Spin Miniprep Kit (Qiagen) and the mutated regions were confirmed by Sanger sequencing.

The 35SRbz‐CSDaV plasmid was also used as template to PCR amplify the gRNA and sgRNA regions using a forward primer containing the T7 promoter sequence and a reverse primer containing poly(A) (Table S1). The PCR products were employed as templates for *in vitro* transcription reactions using the T7 mMESSAGE mMACHINE Kit (Ambion, Austin, TX, USA), according to the manufacturer’s instructions. The capped transcripts (1 µg) were used for *in vitro* translation in a 25‐μL WGE reaction (Promega) in the presence of biotinylated lysine tRNA‐complex (Promega), according to the manufacturer’s instructions. Translation reactions were incubated for 2 h at 25 °C. Translation products were separated by 12.5% SDS‐PAGE and transferred to nitrocellulose membrane. The biotin‐labelled proteins were detected using chemiluminescent substrate (Promega), according to the manufacturer’s instructions.

For *in vivo* analysis, the mutant constructs were introduced into *A. tumefaciens* (GV3101) and agroinfiltrated on 3‐week‐old *N. benthamiana* plants, as described previously. Agroinfiltrated leaves were collected at 2 dpi for total RNA, virion and total protein extractions. Total RNA was checked by northern blot, virions were checked by SDS‐PAGE and TEM, as described above, and total protein extracts were checked by western blot.

For CSDaV CP antibody generation, the peptide C‐GPAPSRDDRVDRQP (Pep‐CSDaV‐CP) was synthesized by Biomatik (Wilmington, DE, USA) based on the CP amino acid sequences (amino acids 1999–2012). Polyclonal anti‐CSDaV‐CP purified antibodies were produced and supplied by Biomatik. For immunoblot analyses of virion CPs, total proteins were extracted from 100 mg of plant material in 100 mm Tris‐HCl (pH 7.5), 100 mm ethylenediaminetetraacetic acid (EDTA) (pH 8.0), 5 mm dithiothreitol, 150 mm NaCl and 0.1% (v/v) Triton X‐100 buffer. Proteins were separated on SDS‐PAGE gels and transferred to nitrocellulose membranes. CSDaV CPs were detected by immunoblotting using the anti‐CSDaV‐CP antibody. Goat anti‐rabbit IgG‐HRP conjugate (Bio‐Rad, Hercules, CA, USA) was used as secondary antibody. Protein bands were visualized using the SuperSignal West Pico Chemiluminescent Substrate (Thermo Fisher Scientific) and ChemiDoc Touch Imaging System (Bio‐Rad, Hercules, CA, USA).

### *In vivo trans*‐protease activity assay

The *trans*‐proteolytic activity of the CSDaV protease was analysed *in vivo* by co‐agroinfiltration of *A. tumefaciens* cells harbouring plasmids for CP‐deficient mutants (used as sources of protease) with the individual CSDaV CPs (substrates) in *N. benthamiana* plants. Coding sequences corresponding to CSDaV CPp25WT (WT clone) and CPp25M‐CS (CPp25 bearing the same mutations as mutant M‐CS, see Fig. 3B) were amplified and In‐Fusion cloned into pEAQ binary vector (Sainsbury *et al.*, 2010). Plasmids pEAQ‐CPp25WT and pEAQ‐CPp25M‐CS were recovered in *E. coli* (DH5α) and introduced into *A. tumefaciens* (GV3101), as described previously. The co‐infiltration combinations used in this assay are shown in Fig. 5A. The *trans*‐protease activity was checked at 2 dpi by immunoblotting of the partially purified virions.

## Supporting information

Fig. S1  Diagram showing the strategy used to construct the 35SRbz‐CSDaV clone. (A) Schematic representation of the genome organization of *Citrus sudden death‐associated virus* (CSDaV) showing the two predicted open reading frames (ORFs) (dark grey shading) and the potentially functional domains (grey boxes): MT, methyltransferase; PRO, protease; HEL, helicase; RdRP, RNA‐dependent RNA polymerase; CP, capsid protein and p16. (B) Map of the pJL89 binary vector used as backbone for the 35SRbz‐CSDaV construction. The positions of the duplicated 2 × 35S promoter, HDV‐Rbz (hepatitis delta virus ribozyme) and NOS (nopaline synthase terminator), as well as other features (oriV, origin of replication; KanR, coding region for kanamycin resistance; trfA, coding region for *trans*‐acting replication protein), are indicated. (C) Two genomic fragments (I and II) overlapping the complete genome of CSDaV were polymerase chain reaction (PCR) amplified and sequentially inserted into pJL89 by In‐Fusion cloning. Arrows indicate the binding positions of the primers (Table S1). Blue and green indicate complementarity to CSDaV fragments I and II, respectively; red indicates complementarity to the pJL89 vector. (D) Electrophoretic pattern of the linearized plasmids and CSDaV fragments I and II (top gels) and electrophoretic pattern of the 35SRbz‐CSDaV clones (1, 2 and 3) digested with *Rsr*II restriction enzyme to confirm the insertion of the full‐length CSDaV sequence into the pJL89 vector. M, 1‐kb plus ladder. L, linearized.

Table S1  Primers used for the construction and analyses of full‐length cDNA clones of *Citrus sudden death‐associated virus* (CSDaV). The purpose of each primer is presented.
