# Supplementary figures and images for: Rescue of Citrus sudden death‐associated virus in Nicotiana benthamiana plants from cloned cDNA: insights into mechanisms of expression of the three capsid proteins
Source: Mol Plant Pathol. 2019 Jan 29;20(5):611–25. doi: 10.1111/mpp.12780 (PMC6637869; doi:10.1111/mpp.12780)

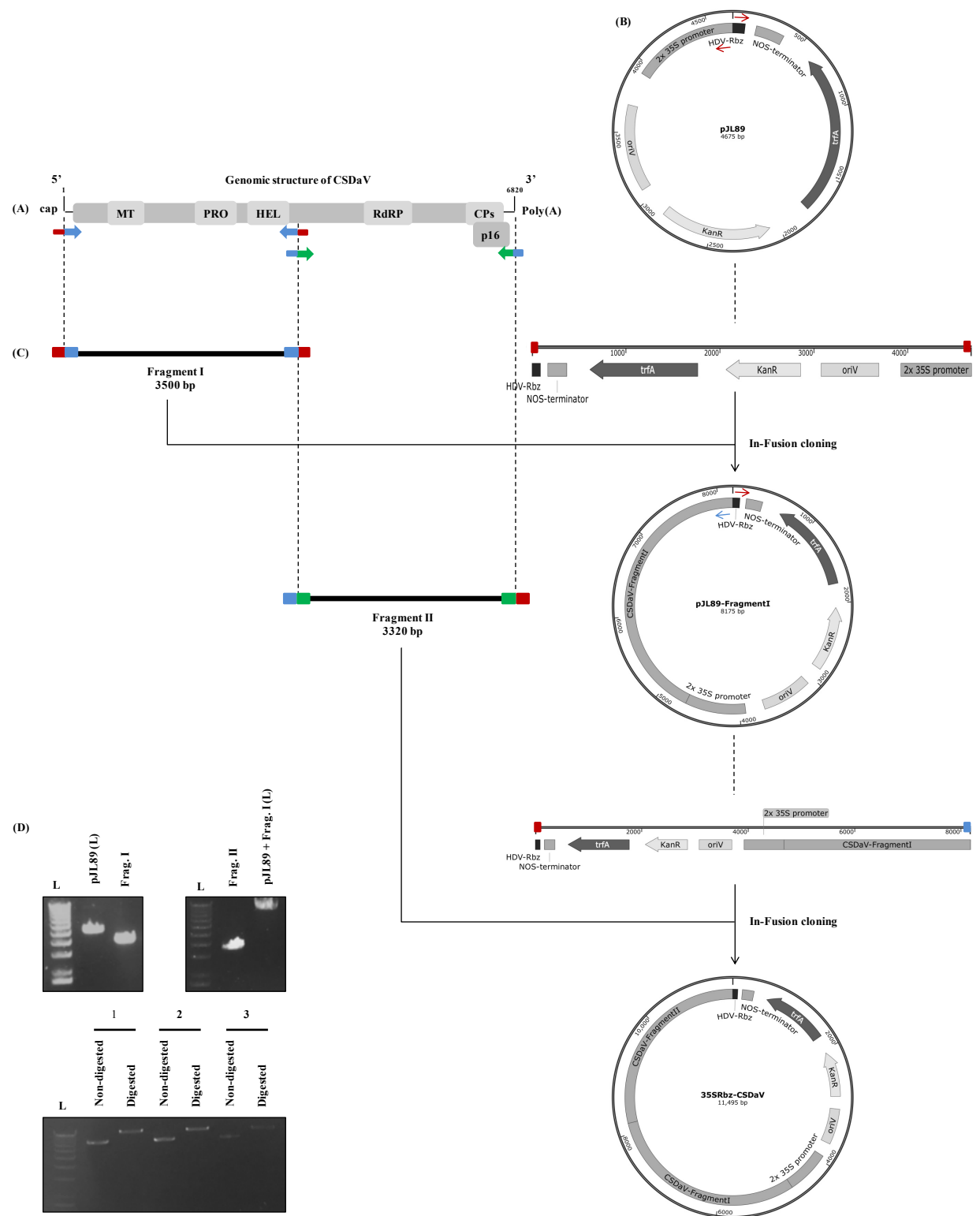

**Fig. S1**

Supplement: Supplementary file 1 — Fig. S1 Diagram showing the strategy used to construct the 35SRbz‐CSDaV clone. (A) Schematic representation of the genome organization of Citrus sudden death‐associated virus (CSDaV) showing the two predicted open reading frames (ORFs) (dark grey shading) and the potentially functional domains (grey boxes): MT, methyltransferase; PRO, protease; HEL, helicase; RdRP, RNA‐dependent RNA polymerase; CP, capsid protein and p16. (B) Map of the pJL89 binary vector used as backbone for the 35SRbz‐CSDaV construction. The positions of the duplicated 2 × 35S promoter, HDV‐Rbz (hepatitis delta virus ribozyme) and NOS (nopaline synthase terminator), as well as other features (oriV, origin of replication; KanR, coding region for kanamycin resistance; trfA, coding region for trans‐acting replication protein), are indicated. (C) Two genomic fragments (I and II) overlapping the complete genome of CSDaV were polymerase chain reaction (PCR) amplified and sequentially inserted into pJL89 by In‐Fusion cloning. Arrows indicate the binding positions of the primers (Table S1). Blue and green indicate complementarity to CSDaV fragments I and II, respectively; red indicates complementarity to the pJL89 vector. (D) Electrophoretic pattern of the linearized plasmids and CSDaV fragments I and II (top gels) and electrophoretic pattern of the 35SRbz‐CSDaV clones (1, 2 and 3) digested with RsrII restriction enzyme to confirm the insertion of the full‐length CSDaV sequence into the pJL89 vector. M, 1‐kb plus ladder. L, linearized. [file MPP-20-611-s001.pdf]
