# Supplementary material for: Rescue of Citrus sudden death‐associated virus in Nicotiana benthamiana plants from cloned cDNA: insights into mechanisms of expression of the three capsid proteins
Source: Mol Plant Pathol. 2019 Jan 29;20(5):611–25. doi: 10.1111/mpp.12780 (PMC6637869; doi:10.1111/mpp.12780)
Supplement: Supplementary file 2 — Table S1 Primers used for the construction and analyses of full‐length cDNA clones of Citrus sudden death‐associated virus (CSDaV). The purpose of each primer is presented. [file MPP-20-611-s002.pdf]

Table S1

Primer	Sequence (5'-3')	Purpose
CSDaV-VF	GGGTCGGCATGGCATCTC	Inverse PCR of the pJL89 vector
CSDaV-VR	CCTCTCCAAATGAAATGAACTTCCTATATAGAGGA	
CSDaV-FIF	GTTCATTTCATTTGGAGAGGGTCCCCTGTGATCGTCTCTCC	Amplification of fragment I of the CSDaV
CSDaV-FIR	TGGAGATGCCATGCCGACCCGGGAGACCAGTAATGGTTTTCCACT	
CSDaV-VIR	GGGAGACCAGTAATGGTTTTCCACTC	Inverse PCR of the pJL89 vector after insertion of fragment I
CSDaV-FIIF	AAAACCATTACTGGTCTCCCGGCCTCAGAAGCCTGGCG	Amplification of fragment II of the CSDaV
CSDaV-FIIR	TGGAGATGCCATGCCGACCCTTTTTTTTTTTTTTTATTAAATAATAAAGAAAAACGGTCTTTGGATCGAC	
CSDaV-midIF	TGGACAGATCTGTGACCTCTTCCTCT	Detection of CSDaV (fragment I) and Sanger sequencing
CSDaV-midIR	TCAGATGATGGGGAGGAGAGCTGAT	
CSDaV-midIIF	TGGTTCCACAATGAGTTCCCAAAGGC	Detection of CSDaV (fragment II) and Sanger sequencing
CSDaV-midIIR	CAATTCACCTTGTAGCAGAGTGGTGTC	
PJL89F	AAGGGATGACGCACAATCCCACTATC	Sanger sequencing
PJL89R	ATCGGGGAAATTCGAGCTCTCCCTTA	
CSDaV-5UTRF	CCCTCCAGCCGGAAAGATATTTTTGC	Sanger sequencing
CSDaV-3UTRR	AGAAAAACGGTCTTTGGATCGACCGG	
CSDaV-IR	GGGAGACCAGTAATGGTTTTCCACTC	Sanger sequencing
CSDaV-IIF	AGGAGATCCGTCCGTCTGATCCATAT	
CSDaV-CPqPCRF	TCTTGCTGCAGCCTTCTCCA	RT-qPCR
CSDaV-CPqPCRR	ACAGGACCGCCAACAGTGAA	RT-qPCR
CSDaV-CPF	GCCATCTACACCACACTCTC	Make probe for Northern blot
CSDaV-CPR	TTGGAGTAGACGGAGTAGGA	
CSDaV-RdRPF	TCCATGCAGGCAACACTCCA	
CSDaV-RdRPR	TTTCAAGGACGACCGCCTCA	
M-AUG1F	CCATTCGTACAATCTGACACTCTCCTGCCTCTCAC	Site directed mutagenesis
M-AUG1R	GTGTCAGATTGTACGAATGGTACTTGAAAGTGCTG	
M-AUG2F	CTCGCTTTCTCCTTAGCCAGCGATGCCC	Site directed mutagenesis

M-AUG2R	AAG<br>CTGGCTAAGGAGAAAGCGAGGGAGGTG<br>GAGAAG	
M-KCAUG1F<br>M-KCAUG1R	CTTTCAAGCTTTCTCCATGGAATCTGAC<br>ACTCTCC<br>CCATGGAGAAAGCTTGAAAGTGCTGAA<br>GCAATTCA	Site directed mutagenesis
M-KCAUG2F<br>M-KCAUG2R	TCGTACCATTCATGCCCAGCGATGCCCA<br>AGCA<br>GCTGGGCATGAATGGTACGAGGGAGGT<br>GGAGAAG	Site directed mutagenesis
M-CSF<br>M-CSR	CTCTCACTGTTGTTTCCTCTGCTCCCATC<br>CTCACAC<br>AGAGGAAACAACAGTGAGAGGCAGGA<br>GAGTGTCAG	Site directed mutagenesis
M-MboxF<br>M-MboxR	GGTGAACTACTTCAGCACTTTCAAGTAC<br>CATTCAT<br>AAGTGCTGAAGTAGTTCACCTTGTAGCA<br>GAGTGGT	Site directed mutagenesis
CPp25-FragF<br>CPp25-21-FragR<br>CPp25-FragF	GTTCATTTCATTTGGAGAGGATGCAATC<br>TGACACTCTCCTGC<br>TGGAGATGCCATGCCGACCCCTAAGCG<br>AGAAGATTACCGGAGG<br>GTTCATTTCATTTGGAGAGGATGGCCAG<br>CGATGCC	Amplification of CPp25 and CPp21
pEAQ-F<br>pEAQ-R	TCGAGGCCTTTAACTCTGGTTTCATTAA<br>ATT<br>TCGCGAATTTGGGCAGAATATACAGAA<br>G	Inverse PCR of the pEAQ vector
GSP1<br>GSP2<br>GSP3<br>Abridged anchor primer	TTGGAGAAGGCTGCAGCAAGAG<br>CAGACCTCCAGCTCAACAGATGTGA<br>TCAGCATGTCGGTAGTTGGCCATGA<br>GGC CAC GCG TCG ACT AGT AGC GGI<br>IGG GII GGG IIG	5'RACE of the sgRNA
T7_sgRNAF<br>T7_gRNAF<br>CSDaV_3'endR	TAATACGACTCACTATAGGGAAGTACC<br>ATTCATGCAATCTGACAC<br>TAATACGACTCACTATAGGGGTCCCCTG<br>TGATCGTCTCTCCCGCC<br>TTTTTTTTTTTTTTTATTAAAT<br>AATAAAGAAAAACGGTCTTTGGATCGA<br>C	sgRNA and full gRNA amplification
